# Intrawound Vancomycin Powder Reduces Deep Surgical Site Infection in Tibial Plateau and Pilon Fractures: A Systematic Review and Meta-Analysis

**DOI:** 10.7759/cureus.115984

**Published:** 2026-09-09

**Authors:** Abdullah Jumaa Hadid, Bcher Chkir, Ahmad Wanli, Mhd Hadi Abou Samara, Mohamed Ismail Sheakh Bakri

**Affiliations:** 1 Orthopaedics, NHS Wales Shared Services Partnership, Cardiff, GBR; 2 Internal Medicine, NHS Wales Shared Services Partnership, Cardiff, GBR; 3 Internal Medicine, National Health Service Wales, Newport, GBR; 4 Internal Medicine, Mater Dei Hospital, Valleta, MLT; 5 Orthopaedics, Mater Dei Hospital, Valleta, MLT

**Keywords:** fracture fixation, infection prevention, intrawound antibiotic, meta-analysis, orthopaedic trauma, surgical site infection, systematic review, tibial pilon fracture, tibial plateau fracture, vancomycin powder

## Abstract

Deep surgical site infection (SSI) after operative fixation of tibial plateau and pilon fractures remains a common complication despite modern surgical protocols. A systematic review and meta-analysis was performed to determine whether intrawound vancomycin powder reduces deep SSI in these fractures. PubMed, Cochrane CENTRAL, and Scopus were searched from inception to February 20, 2026. Our study identified that vancomycin powder was associated with reduced deep SSI (odds ratio (OR) 0.53; 95% confidence interval (CI) 0.35-0.80; p = 0.002; I² = 0%), with an infection rate of 4.9% versus 9.7%. The effect was driven by gram-positive deep infections (OR 0.32; 95% CI 0.14-0.73; p = 0.007). No effect was observed on superficial SSI (OR 1.17; p = 0.66). Results were robust across all sensitivity analyses including leave-one-out, fixed-effect, Peto, and subgroup analyses by study design and fracture risk level. The certainty of evidence was moderate for deep SSI and high for gram-positive deep SSI (Grading of Recommendations, Assessment, Development and Evaluations (GRADE)). Intrawound vancomycin powder appears to be a safe, low-cost intervention that may be considered for routine use in operative fixation of tibial plateau and pilon fractures.

## Introduction and background

Tibial plateau and pilon fractures are high-energy periarticular injuries that frequently require open reduction and internal fixation (ORIF) [1,2]. Despite advances in staged surgical protocols and perioperative systemic antibiotic prophylaxis, deep surgical site infection (SSI) remains a common complication, with reported rates of 5-16% [3-5]. Deep infection after fixation of these fractures often necessitates multiple return surgeries, prolonged intravenous antibiotic therapy, and may ultimately compromise fracture union, resulting in significant patient morbidity and healthcare costs. The most common causative organisms are gram-positive bacteria, particularly *Staphylococcus aureus*, which account for over half of all implant-related infections in orthopaedic trauma [6].

Intrawound vancomycin powder, applied over fixation hardware at wound closure, delivers high local antibiotic concentrations while minimising systemic exposure [7]. Meta-analyses in spine surgery have demonstrated reduced SSI rates with this approach [8]. A survey across 28 orthopaedic trauma centres found significant variability in vancomycin powder use, highlighting the need for evidence synthesis [9]. The VANCO trial--a multicentre randomised controlled trial (RCT) of 980 patients with high-risk tibial plateau and pilon fractures--found that intrawound vancomycin reduced gram-positive infections (risk difference −3.7%; p = 0.02) but the primary outcome of all-cause deep SSI narrowly missed significance (p = 0.06) [10]. A broader meta-analysis by Ma et al. examined intrawound antibiotics across all pelvic and lower-limb fractures but found a non-significant pooled effect (OR 0.77; 95% CI 0.52-1.13; p = 0.18), possibly because the heterogeneous fracture population diluted the treatment signal [11].

A systematic review and meta-analysis was therefore performed to determine whether intrawound vancomycin powder reduces deep SSI specifically in adults undergoing operative fixation of tibial plateau and pilon fractures. Secondary objectives included evaluating the effect on gram-positive infections, assessing safety outcomes, and exploring consistency across fracture risk levels.

The study protocol was registered with PROSPERO (CRD420261335452).

## Review

Methods

This systematic review and meta-analysis was performed in accordance with the Preferred Reporting Items for Systematic Reviews and Meta-Analyses (PRISMA) 2020 statement [12] and the Cochrane Handbook for Systematic Reviews of Interventions [13].

Eligibility Criteria

Eligible studies enrolled adults (≥18 years) with tibial plateau or pilon fractures treated with ORIF, compared intrawound vancomycin powder at wound closure versus no vancomycin, reported at least 1 prespecified outcome, and were RCTs or comparative cohort studies. Studies using mixed antibiotic interventions where vancomycin-only data could not be isolated were excluded. One study (Miles 2023) was excluded because 29% of its treatment group received combined vancomycin and tobramycin [14].

Search Strategy

PubMed, Cochrane CENTRAL, and Scopus were searched from inception to February 20, 2026, with no restrictions (Table 1). ClinicalTrials.gov and reference lists of included studies and a prior meta-analysis [11] were also searched. Two reviewers independently screened titles, abstracts, and full texts. Data extraction used a standardised form; all values were verified against original publications. Three studies (Qadir 2021 [15], Zingas 2023 [16], and the VANCO trial [10]) involved the University of Maryland with no patient overlap: Qadir’s cohort predated the VANCO trial, and Zingas excluded all VANCO-eligible patients. For Qadir 2021, tibial plateau and pilon data were derived by subtracting calcaneus data from reported totals. The subtraction approach was valid because the vancomycin arm had zero events across all fracture types in the original cohort (0/35), guaranteeing zero events in any subset.

**Table 1:** Database search strategies

Database	Search String	Fields	Filters	Results
PubMed	("tibial plateau" OR "tibial plafond" OR "tibial pilon" OR "pilon fracture" OR "plateau fracture" OR "periarticular tibia" OR "peri-articular tibia" OR "distal tibia" OR "proximal tibia") AND (vancomycin OR "vancomycin powder" OR "intrawound vancomycin" OR "topical vancomycin" OR "local vancomycin" OR "intrawound antibiotic" OR "topical antibiotic" OR "local antibiotic powder" OR "antibiotic powder")	All fields	None	27
Cochrane CENTRAL	("tibial plateau" OR "tibial plafond" OR "tibial pilon" OR "pilon fracture" OR "plateau fracture" OR "periarticular tibia" OR "peri-articular tibia" OR "distal tibia" OR "proximal tibia") AND (vancomycin OR "vancomycin powder" OR "intrawound vancomycin" OR "topical vancomycin" OR "local vancomycin" OR "intrawound antibiotic" OR "topical antibiotic" OR "local antibiotic powder" OR "antibiotic powder")	Title, Abstract, Keywords	None	13
Scopus	TITLE-ABS-KEY("tibial plateau" OR "tibial plafond" OR "tibial pilon" OR "pilon fracture" OR "plateau fracture" OR "periarticular tibia" OR "peri-articular tibia" OR "distal tibia" OR "proximal tibia") AND TITLE-ABS(vancomycin OR "vancomycin powder" OR "intrawound vancomycin" OR "topical vancomycin" OR "local vancomycin" OR "intrawound antibiotic" OR "topical antibiotic" OR "local antibiotic powder" OR "antibiotic powder")	Population: TITLE-ABS-KEY; Intervention: TITLE-ABS	None	61
ClinicalTrials.gov	Search 1: "vancomycin tibial plateau" Search 2: "vancomycin pilon" Search 3: "vancomycin tibial fracture"	All fields	None	4 trials; 0 with eligible completed results
Manual search	Reference lists of all included studies and Ma et al. 2022 meta-analysis [11]	N/A	N/A	0 additional
Total				101 database records + 4 registry entries

Outcomes

The primary outcome was deep SSI or fracture-related infection (FRI). Deep SSI was defined by modified Centres for Disease Control and Prevention (CDC)/National Healthcare Safety Network (NHSN) criteria in the VANCO trial, standard CDC criteria in Singh 2015 [17], Qadir 2021 [15], and Zingas 2023 [16], and by international FRI consensus criteria [18] in Wang 2023 [19]. All definitions required surgical treatment. Pooling across fracture risk levels was considered appropriate because the intervention, comparator, outcome definitions, and biological mechanism were consistent across studies. Secondary outcomes included gram-positive deep SSI, superficial SSI, nonunion, wound dehiscence, and nephrotoxicity.

Quality Assessment

Risk of bias was assessed independently by two reviewers using the Cochrane Risk of Bias 2 (RoB 2) tool [20] for the RCT and the Risk of Bias in Non-Randomised Studies of Interventions (ROBINS-I) tool [21] for retrospective studies. Disagreements were resolved by consensus; all were within one level and did not affect overall study judgments. Publication bias was assessed by funnel plot visual inspection; formal tests were not performed for fewer than 10 studies [13]. Certainty of evidence was assessed using the GRADE framework.

Statistical Analysis

Odds ratios (OR) with 95% confidence intervals (CI) were pooled using a DerSimonian and Laird random-effects model. Fixed-effect and Peto methods served as sensitivity analyses. A continuity correction of 0.5 was applied for zero-event studies. Heterogeneity was assessed using Cochran Q and I² (>25% = meaningful). Two-sided p <0.05 was used for effect tests; p <0.10 for interaction tests. Analyses used R version 4.3.3 (meta package) with Python 3.12 for supplementary calculations. Fracture classification followed the OTA/AO system [22]. Prespecified subgroup analyses examined study design (RCT versus retrospective) and fracture risk level. Sensitivity analyses included leave-one-out, exclusion of Qadir 2021 (derived data), and exclusion of Singh 2015 (n = 10 vancomycin patients). The forest plots display both Mantel-Haenszel common-effect and DerSimonian-Laird random-effects estimates. The inverse-variance fixed-effect estimate (OR 0.53) reported in the text is identical to the random-effects estimate because between-study variance (τ²) was zero.

Results

Study Selection and Characteristics

Database searches identified 101 records. After removal of 33 duplicates, 68 unique records were screened. Fifteen full-text articles were assessed; 10 were excluded. Five studies met all criteria (Figure 1).

**Figure 1:**
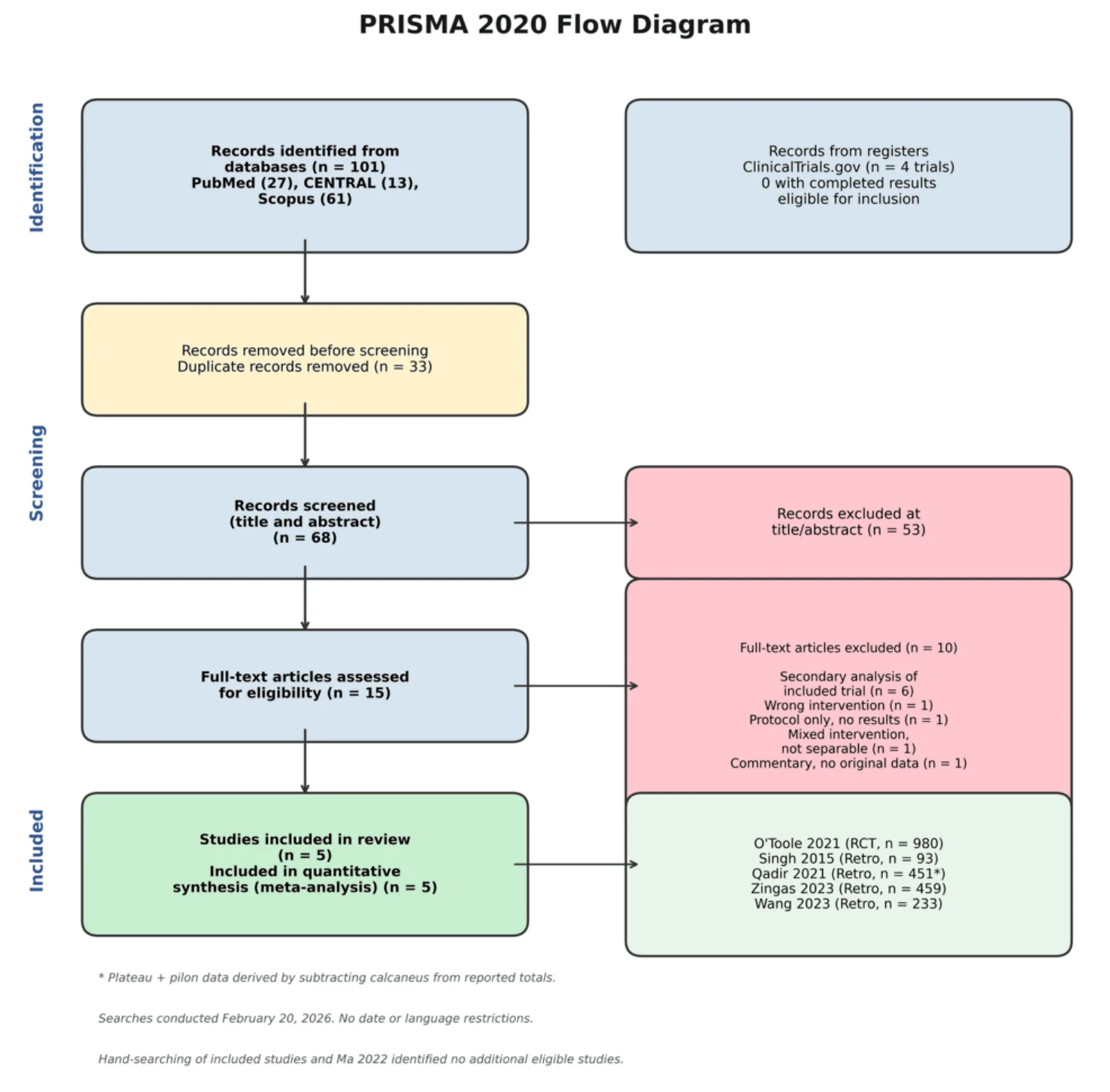
PRISMA flow diagram

Ten full-text articles were excluded (Table 2): one mixed vancomycin-tobramycin study with inseparable data [14], the VANCO trial nephrotoxicity substudy [23], a Bayesian reanalysis [24], a German-language commentary [25], a bacteriological analysis [26], a generalisability analysis [27], an analysis of drain use [28], and a superficial-versus-deep infection analysis [29]. Two further reports are cited below as context rather than as screened records: a study of vancomycin applied at initial debridement rather than at definitive closure [30], and the protocol for the ongoing TOBRA trial [31]. The two remaining exclusions were a study evaluating only the safety of tobramycin [32] and the VANCO trial protocol [33].

**Table 2:** Full-text screening

First Author	Year	Title	Study Design	Population / Fracture Type	Sample Size (V / C)	Study Family (independent or VANCO trial?)	Reviewer 1 (Include/Exclude)	Reviewer 2 (Include/Exclude)
O'Toole [33]	2017	Local antibiotic therapy to reduce infection... (VANCO study protocol)	RCT protocol	High-risk plateau/pilon	~980	VANCO trial	Exclude	Exclude
Stengel [25]	2022	Local vancomycin powder during surgical fixation of tibial plateau and plafond fractures	Commentary	Plateau/plafond	Unknown	Unknown	German-language commentary on the VANCO trial; no original patient data.	Exclude
Schrank [32]	2025	Safety of topical tobramycin powder during operative treatment of fractures	Prospective observational	Tibial fractures	Unknown	TOBRA trial	Exclude	Exclude
Patterson [27]	2024	The VANCO trial findings are generalizable to a North American trauma registry	Secondary analysis	Plateau/pilon	VANCO + registry	VANCO trial	Exclude	Exclude
Patterson [29]	2023	Do superficial infections increase the risk of deep infections in tibial plateau and plafond fractures?	Secondary analysis	Plateau/pilon	VANCO trial	VANCO trial	Exclude	Exclude
Qadir/Carlini [15]	2021	Vancomycin powder use in fractures at high risk of surgical site infection	Retrospective cohort	Plateau, pilon, calcaneus	451 "plateau+pilon subset; 583 total cohort"	Independent	INCLUDE	INCLUDE
Singh/Bauer [17]	2015	SSI in high-energy peri-articular tibia fractures with intra-wound vancomycin powder	Retrospective pilot	High-energy plateau/pilon	93	Independent	INCLUDE	INCLUDE
O'Hara [24]	2023	Application of Bayesian methods to help interpret the VANCO trial results	Secondary analysis	Plateau/pilon	VANCO trial	VANCO trial	Exclude	Exclude
Miles [14]	2023	Topical antibiotic powder and nonunion risk in tibial plateau and pilon fractures	Secondary analysis	Plateau/pilon	VANCO trial	VANCO trial	Exclude	Exclude
Joshi [26]	2024	Does topical vancomycin change bacteriology? VANCO trial analysis	Secondary analysis	Plateau/pilon	VANCO trial	VANCO trial	Exclude	Exclude
O'Toole [10]	2021	Effect of intrawound vancomycin powder in operatively treated high-risk tibia fractures: a randomized clinical trial	Multicenter RCT	High-risk plateau/pilon	481/499	VANCO trial PRIMARY	INCLUDE	INCLUDE
Boissonneault [28]	2025	Are drains associated with infection after operative fixation of high-risk tibial plateau and pilon fractures?	Secondary analysis	Plateau/pilon	VANCO trial	VANCO trial	Exclude	Exclude
Zingas [16]	2023	The use of vancomycin powder in tibial plateau fractures at lower risk of infection	Retrospective cohort	Low-risk plateau	459 (V vs C)	Independent	INCLUDE	INCLUDE
Wang [19]	2023	Intrawound vancomycin reduces FRI in high-risk tibial plateau fractures	Retrospective cohort	High-risk plateau	233	Independent	INCLUDE	INCLUDE
O'Toole [23]	2021	Systemic absorption and nephrotoxicity associated with topical vancomycin powder for fracture surgery	Prospective observational substudy	Plateau/pilon	58 (treatment arm)	VANCO trial	Exclude	Exclude

The five studies comprised 2,216 patients (740 vancomycin, 1,476 control; Table 3).

**Table 3:** Characteristics of included studies ᵃ Plateau and pilon data derived by subtracting calcaneus from reported totals; 583 total cohort; ᵇ Excluded all patients meeting VANCO trial high-risk criteria; ᶜ All 16 open fractures in vancomycin group (p < 0.001). CDC: Centres for Disease Control and Prevention; NHSN: National Healthcare Safety Network

Study	Year	Design	Setting	N (V/C)	Population	Intervention	Follow-up	SSI Definition
O'Toole et al. (VANCO trial)[10]	2021	Multicentre open-label RCT	36 US trauma centres	980 (481/499)	High-risk tibial plateau and pilon. Staged with ex-fix >3 d, Gustilo I/II/IIIA open, or compartment syndrome with fasciotomy.	1 g vancomycin powder over implants at closure. Both groups: standard IV prophylaxis (cefazolin ~87%).	182 days; 91% expected person-time	Modified CDC/NHSN; deep = surgically treated; centrally adjudicated, blinded.
Singh et al. [17]	2015	Single-centre retrospective	Vanderbilt, USA	93 (10/83)	High-risk tibial plateau and pilon. High-energy injuries requiring staged fixation (definitive ≥5 d post-injury).	1 g vancomycin powder at definitive fixation, surgeon discretion. IV cefazolin 1 g pre-op + q8h × 24 h.	Minimum 6 months	CDC criteria; deep = requiring operative I&D.
Qadir et al. ᵃ [15]	2021	Single-centre retrospective	University of Maryland, USA	451 ᵃ (29/422)	High-risk tibial plateau, pilon, and calcaneus requiring staged fixation. Plateau + pilon data extracted for this review.	1 g vancomycin powder over fixation device at closure. IV cefazolin pre-op + 24 h post-op.	Minimum 6 months	CDC criteria; deep = operative debridement deep to subcutaneous layer.
Zingas et al. ᵇ [16]	2023	Single-centre retrospective	University of Maryland, USA	459 (115/344)	Low-risk tibial plateau only (OTA/AO 41-B/C). Excluded open fractures, compartment syndrome, and external fixation.	Vancomycin powder at closure; 73% 1 g, 24% 2 g, 2% 0.5 g (surgeon discretion). Standard IV prophylaxis.	Minimum 90 days (median 216–273 d)	CDC criteria; deep = operative debridement. Primary outcome = gram-positive deep SSI.
Wang et al. ᶜ [19]	2023	Two-centre retrospective	Beijing, China	233 (105/128)	High-risk tibial plateau only (AO type B/C). Delayed fixation ≥3 d or Gustilo I/II/IIIA open. Excluded IIIB/IIIC.	1 g vancomycin powder on locking plate and bone before closure. IV cefuroxime pre-op + 3 d post-op.	12 months (3 vanc, 16 ctrl lost before 12 mo; included until loss)	International FRI consensus criteria (Metsemakers [18] 2018 ); ≥1 confirmatory sign or ≥2 positive deep cultures.

One was a multicentre RCT (O’Toole 2021 [10], 36 US trauma centres, n = 980). Four were retrospective: Singh 2015 [17] (Vanderbilt, n = 93), Qadir 2021 [15] (University of Maryland, n = 451), Zingas 2023 [16] (University of Maryland, n = 459), and Wang 2023 [19] (Beijing, China, n = 233). Four enrolled high-risk fractures; Zingas 2023 enrolled low-risk tibial plateau fractures. Wang 2023 had a notable imbalance: all 16 open fractures were in the vancomycin group (p < 0.001), likely biasing toward the null [19].

Primary Outcome

Vancomycin powder was associated with reduced deep infection (OR 0.53; 95% CI 0.35-0.80; p = 0.002; I² = 0%; Figure 2). Deep infection occurred in 36/740 (4.9%) vancomycin patients versus 143/1,476 (9.7%) controls. Heterogeneity was absent (Q = 2.73, df = 4, p = 0.60), although statistical power to detect between-study heterogeneity was limited with only 5 studies. The VANCO trial carried 73.2% of the pooled weight (OR 0.63; 95% CI 0.39-1.02; p = 0.06). All four retrospective studies individually favoured vancomycin. Individual study results are presented in Table 4.

**Figure 2:**
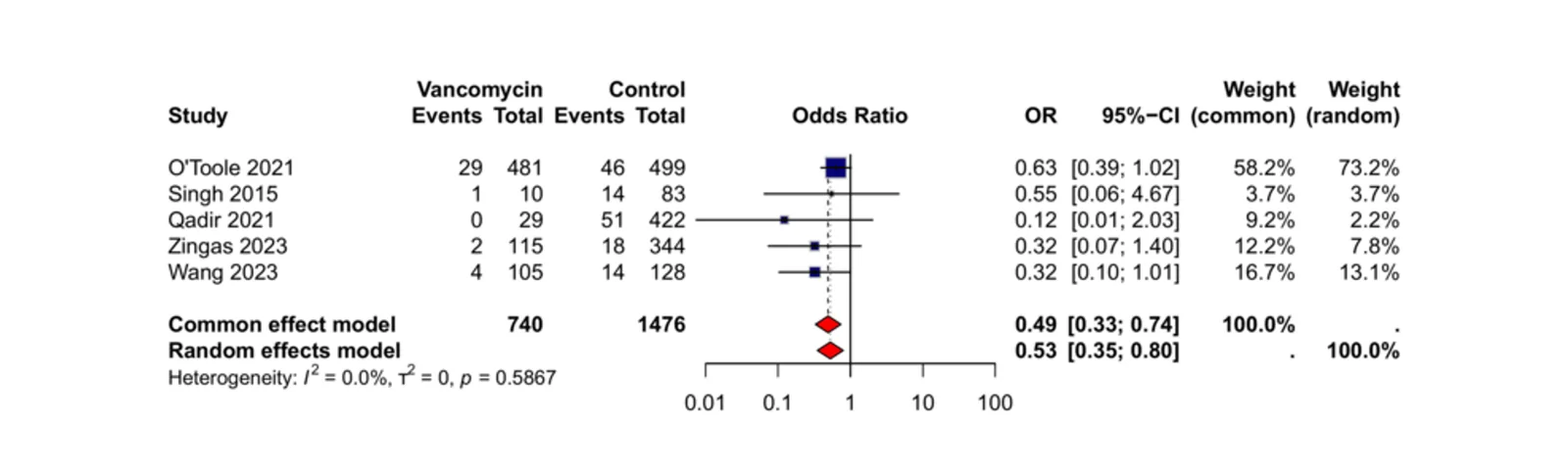
Forest plot for the primary outcome: deep surgical site infection Source studies: O'Toole 2021 [10], Qadir 2021 [15], Zingas 2023 [16], Singh 2015 [17], Wang 2023 [19]

**Table 4:** Individual study results for the primary outcome

Study	Vancomycin events/total	Control events/total	Vancomycin rate	Control rate	OR (95% CI)	Weight
O'Toole 2021 [10]	29/481	46/499	6.0%	9.2%	0.63 (0.39–1.02)	73.2%
Singh 2015 [17]	1/10	14/83	10.0%	16.9%	0.55 (0.06–4.67)	3.7%
Qadir 2021 [15]	0/29	51/422	0%	12.1%	0.12 (0.01–2.03)	2.2%
Zingas 2023 [16]	2/115	18/344	1.7%	5.2%	0.32 (0.07–1.40)	7.8%
Wang 2023 [19]	4/105	14/128	3.8%	10.9%	0.32 (0.10–1.01)	13.1%
Pooled (RE, DL)	36/740	143/1,476	4.9%	9.7%	0.53 (0.35–0.80)	100%

Gram-Positive Deep SSI

Four studies contributed data. Vancomycin was associated with reduced gram-positive deep infection (OR 0.32; 95% CI 0.14-0.73; p = 0.007; I² = 15%; Figure 3), with rates of 2.4% versus 6.8% (68% odds reduction). Gram-negative infections in the VANCO trial were unaffected (OR 1.13; p = 0.78), confirming biological specificity.

**Figure 3:**
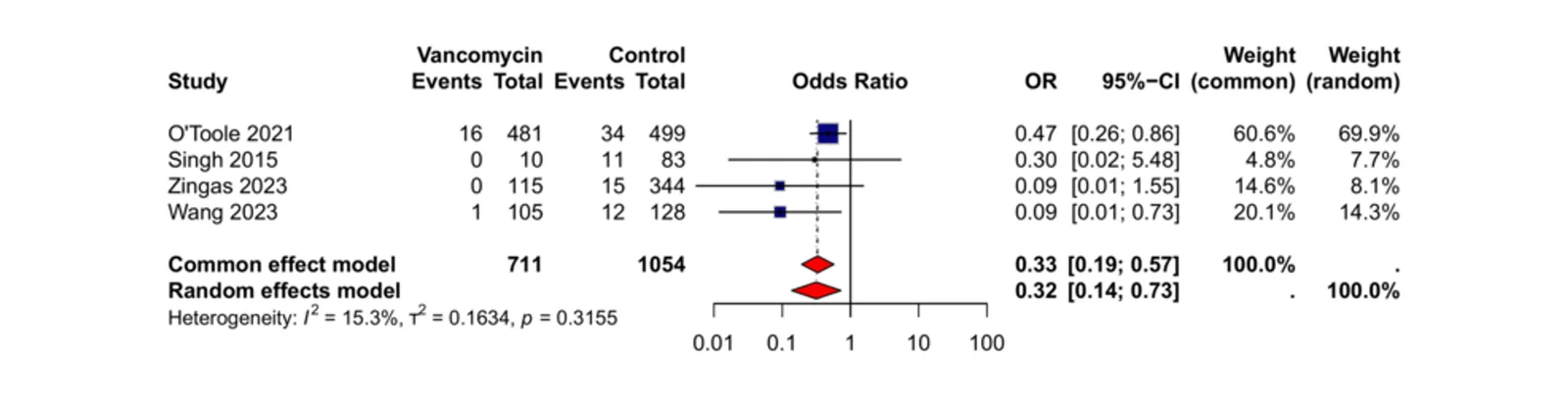
Forest plot for Gram-positive deep surgical site infection Source studies: O'Toole 2021 [10], Zingas 2023 [16], Singh 2015 [17], Wang 2023 [19]

Other Secondary Outcomes

Superficial SSI showed no difference (OR 1.17; 95% CI 0.58-2.36; p = 0.66; 2 studies). O’Toole 2021 alone reported nonunion (5.3% vs 4.1%; p = 0.43) and wound dehiscence (3.9% vs 5.2%; p = 0.42), with no differences [10]. A pharmacokinetic substudy (n = 58) found 96.6% of patients had undetectable serum vancomycin levels with no increase in creatinine [23].

Subgroup and Sensitivity Analyses

Results were robust across all analyses (Figure 4). By study design: RCT OR 0.63 (p = 0.06), retrospective OR 0.32 (p = 0.005); interaction p = 0.15. By fracture risk: high-risk OR 0.55 (p = 0.006), low-risk OR 0.32 (p = 0.13); interaction p = 0.49. Leave-one-out analysis maintained significance throughout (OR range 0.32-0.57). Fixed-effect (OR 0.53; p = 0.002) and Peto (OR 0.52; p = 0.0005) methods were consistent.

**Figure 4:**
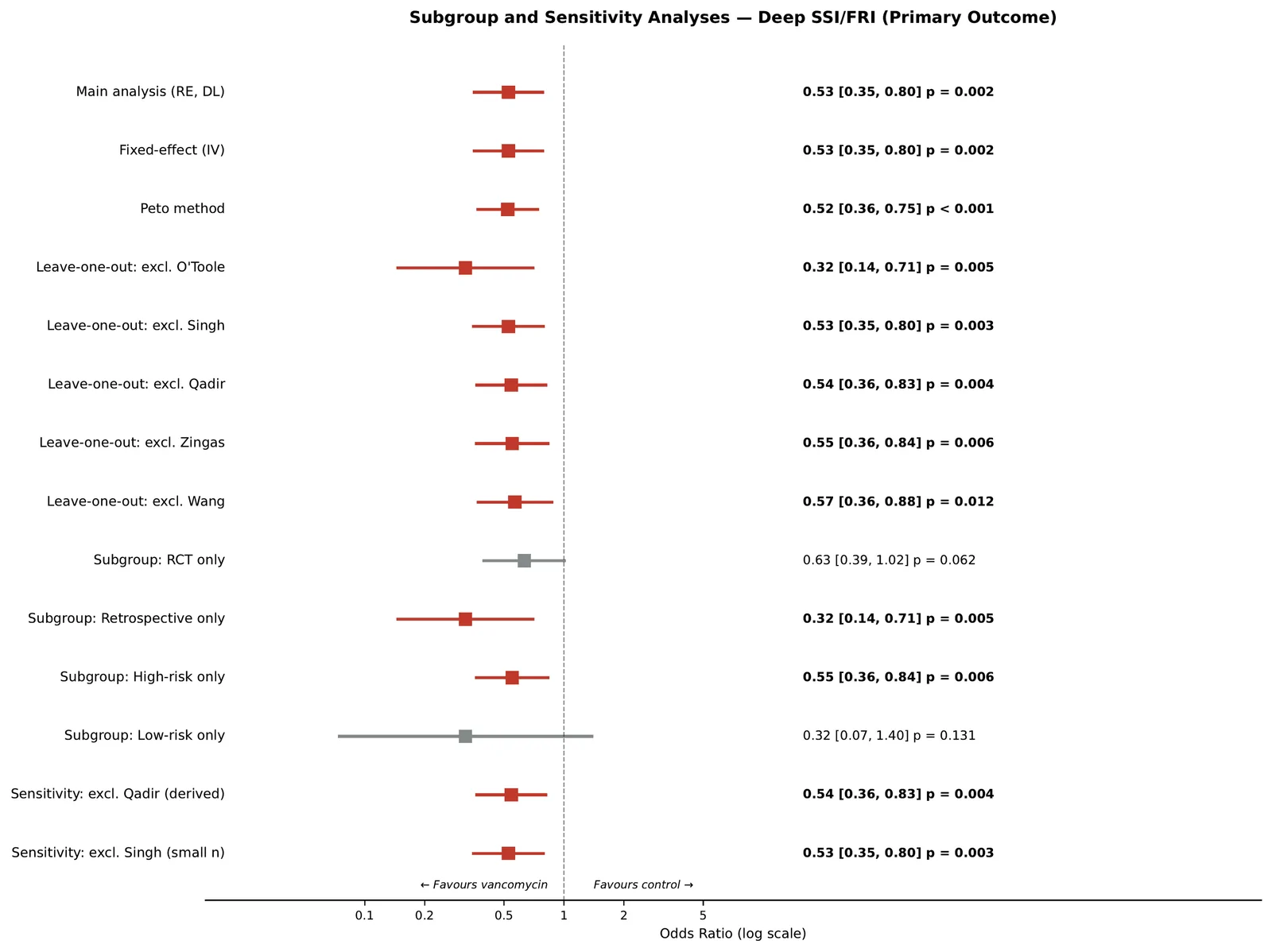
Summary of subgroup and sensitivity analyses for the primary outcome Source studies: O'Toole 2021 [10], Qadir 2021 [15], Zingas 2023 [16], Singh 2015 [17], Wang 2023 [19]

Risk of Bias and Certainty of Evidence

The RoB 2 assessment of O’Toole 2021 yielded “some concerns” (open-label design, post hoc gram-positive framing) (Figure 5) [10]. ROBINS-I yielded “serious” for Singh 2015 and “moderate” for the other three retrospective studies (Figure 6) [15-17,19].

**Figure 5:**
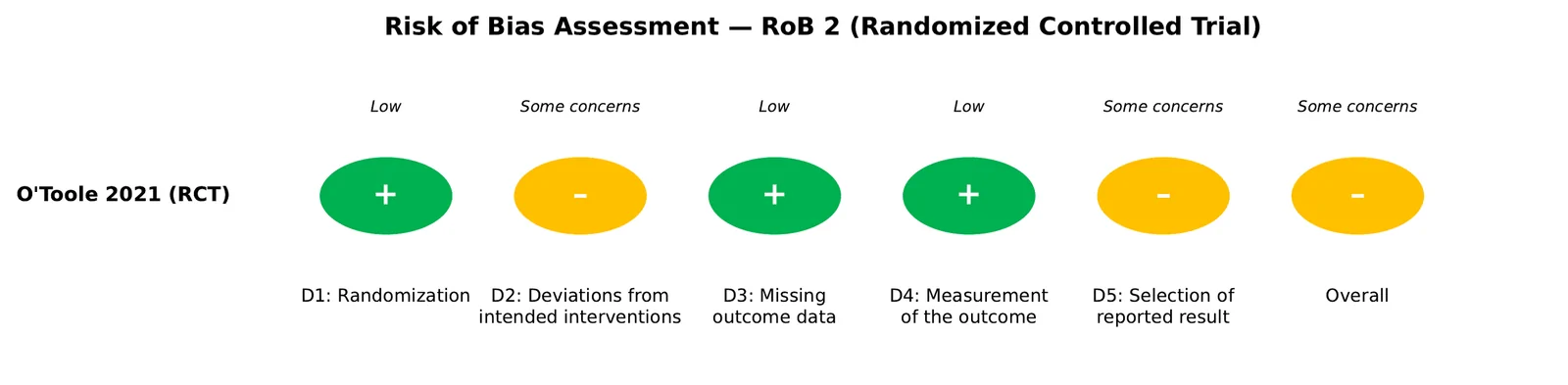
RoB2 assessment Source study: O'Toole 2021 [10]

**Figure 6:**
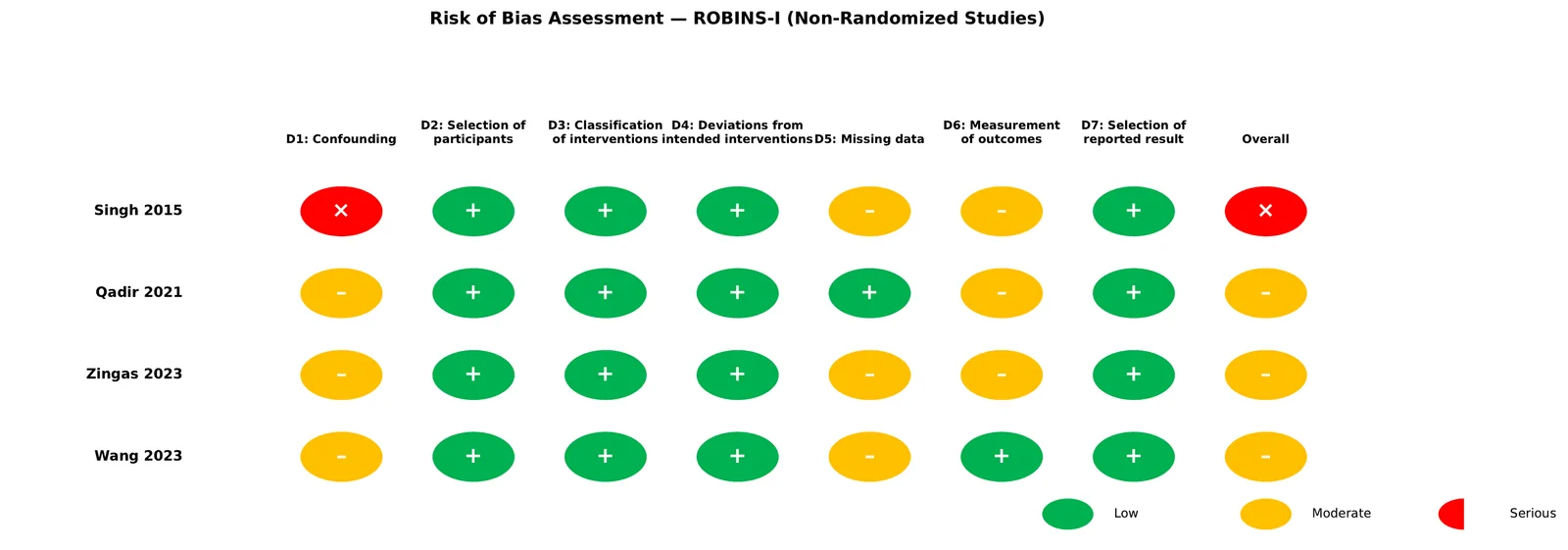
ROBINS-I assessment Source studies: Qadir 2021 [15], Zingas 2023 [16], Singh 2015 [17], Wang 2023 [19]

Singh 2015 carried only 3.7% of the pooled weight [17]. The funnel plot showed no asymmetry (Figure 7).

**Figure 7:**
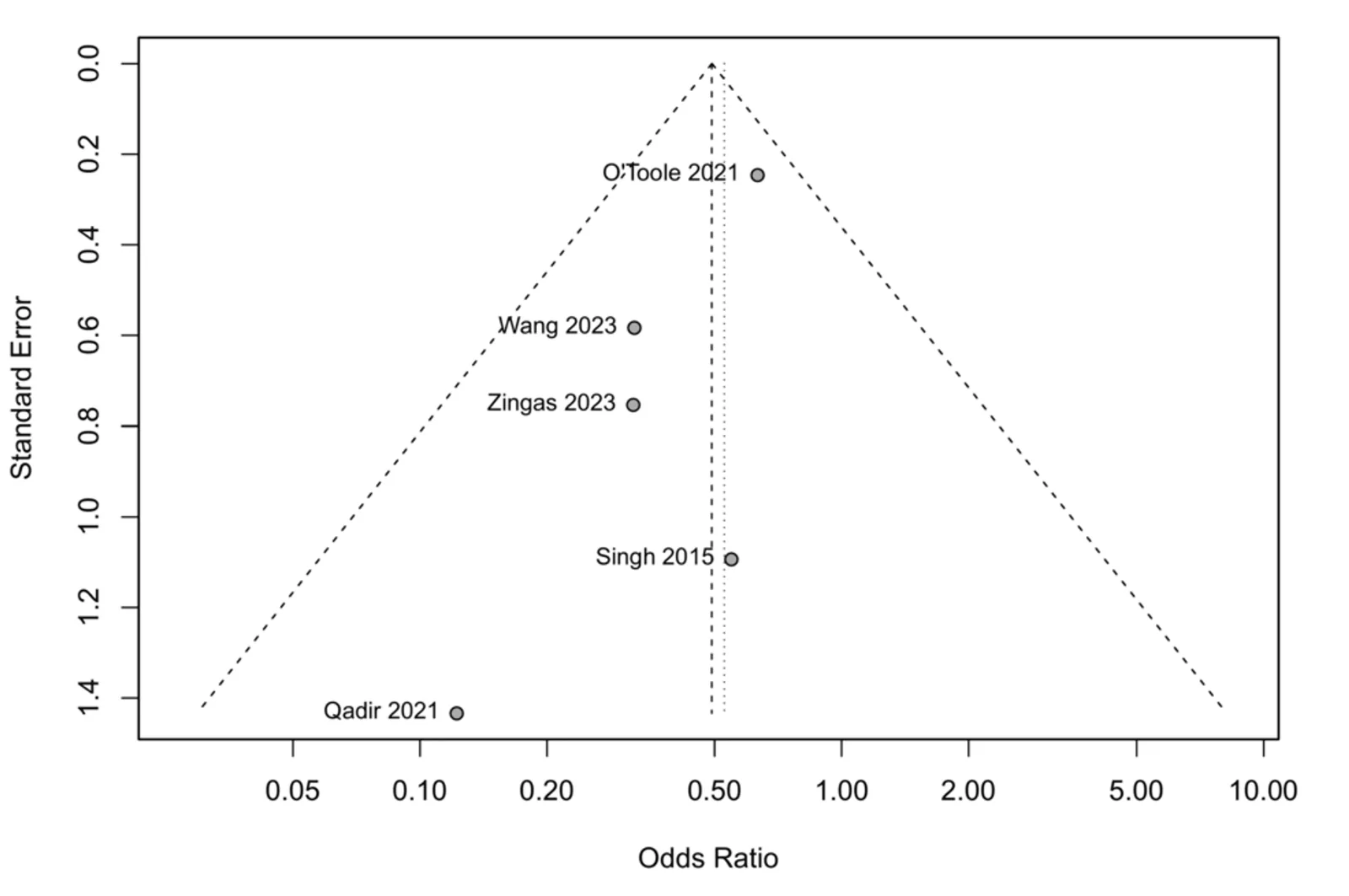
Funnel plot for the primary outcome Source studies: O'Toole 2021 [10], Qadir 2021 [15], Zingas 2023 [16], Singh 2015 [17], Wang 2023 [19]

Using GRADE with a weight-based starting point (RCT = 73% weight), certainty was moderate for deep SSI and high for gram-positive deep SSI. Using a conservative starting point of LOW certainty, ratings would be LOW for deep SSI and MODERATE for gram-positive deep SSI. The GRADE summary of findings is presented in Table 5.

**Table 5:** GRADE summary of findings

Outcome	Studies (patients)	OR (95% CI)	p-value	I²	Vanc rate	Ctrl rate	Certainty	Rationale
Deep SSI	5 (2,216)	0.53 (0.35–0.80)	0.002	0%	4.9%	9.7%	MODERATE	⬇ Risk of bias: open-label RCT, retrospective studies
Gram-positive deep SSI	4 (1,765)	0.32 (0.14–0.73)	0.007	15%	2.4%	6.8%	HIGH	⬇ Risk of bias; ⬆ Large effect + biological specificity
Superficial SSI	2 (963)	1.17 (0.58–2.36)	0.66	0%	3.8%	4.0%	LOW	⬇ Risk of bias; ⬇ Imprecision (wide CI)

Discussion

In this meta-analysis of 5 studies and 2,216 patients, intrawound vancomycin powder was associated with a 47% reduction in deep SSI (OR 0.53; p = 0.002) and a 68% reduction in gram-positive deep infections (OR 0.32; p = 0.007) after operative fixation of tibial plateau and pilon fractures. No effect was observed on superficial SSI, nonunion, or wound dehiscence. Heterogeneity was absent, and results were robust across all sensitivity analyses.

The VANCO trial’s primary outcome fell just short of significance (p = 0.06). A Bayesian reanalysis estimated >98% probability that vancomycin reduces deep SSI (relative risk 0.66; 95% credible interval 0.46-0.98) [24]. Stengel et al. calculated a fragility index of 4, a number needed to treat of 32, and a likelihood of being helped versus harmed of 2.6 [25]. The present meta-analysis resolves this ambiguity with a clearly significant pooled estimate (p = 0.002).

The gram-positive specificity strengthens causal interpretation. Bacteriological analysis of the VANCO trial showed that methicillin-sensitive *Staphylococcus aureus* (MSSA) drove the reduction (1.4% vs 4.8%, p = 0.01), with no emergence of vancomycin-resistant organisms [26]. This pattern was consistent across independent studies.

The effect appeared consistent across fracture severity, with the low-risk subgroup showing a numerically larger effect (OR 0.32) than the high-risk subgroup (OR 0.55), though the interaction was non-significant (p = 0.49). A generalisability analysis found a Tipton index of 0.96 and a reweighted effect (OR 0.46) even larger than the trial estimate [27].

Safety data are reassuring. No increase in nonunion was observed in the VANCO trial [10] or in an independent study (adjusted OR 1.71; p = 0.23) [14]. Negligible systemic absorption was confirmed [23]. A significant drain-vancomycin interaction was identified: drain-only patients had 13% infection versus 3% with drain plus vancomycin (interaction p = 0.048) [28].

The absence of a superficial SSI effect is consistent with intrawound delivery. In the VANCO trial, 92% of deep infections had no preceding superficial infection [29]. Notably, these findings apply to vancomycin at definitive closure; application at initial debridement of open fractures was associated with increased wound complications [30].

The TOBRA trial (NCT04597008) is evaluating whether adding tobramycin provides additional benefit [31], with early safety data showing no acute kidney injury in 97% of patients [32]. The original VANCO trial protocol has been previously published [33].

Limitations

The limitations of this review include the retrospective design of 4 studies, although the RCT dominates at a 73% weight with non-significant interaction by design (p = 0.15); however, this dominance means the pooled estimate is heavily influenced by a single trial and should be interpreted accordingly. The small number of studies limits subgroup power and precludes formal publication bias testing. SSI definitions and follow-up periods varied. Qadir 2021 data were derived from a mixed-fracture cohort [15]. All but one study were US-based.

## Conclusions

Intrawound vancomycin powder at the time of definitive fixation was associated with a 47% reduction in the odds of deep SSI in tibial plateau and pilon fractures, with a 68% reduction in Gram-positive infections. The effect was consistent across study designs, statistical methods and fracture risk levels. No safety concerns were identified. Vancomycin powder appears to be a safe, low-cost intervention that may be considered for routine use in operative fixation of these fractures.
